# Correction

**DOI:** 10.1080/15592294.2025.2554384

**Published:** 2025-09-10

**Article title**: Two novel SIRT1 activators, SCIC2 and SCIC2.1, enhance SIRT1-mediated effects in stress response and senescence

**Authors**: Lucia Scisciola, Federica Sarno, Vincenzo Carafa, Sandro Cosconati, Salvatore Di Maro, Loreta Ciuffreda, Antonella De Angelis, Paola Stiuso, Alessandra Feoli, Gianluca Sbardella, Lucia Altucci, and Angela Nebbioso

**Journal**: *Epigenetics*

**Bibliometrics**: Volume 15, Number 6-7, pages 664-683

**DOI**: https://doi.org/10.1080/15592294.2019.1704349

It has been noted by the authors that Figures 7 and 8 contain errors in the published article. The corrected text for the panels of Figure 7b and the revised image for DOXO + SCIC2.1 in Figure 8a are provided below, embebbed in the full figures. This correction does not affect the description, interpretation, or original conclusions of the article. The authors apologize for any inconvenience caused.

**Figure 7.**
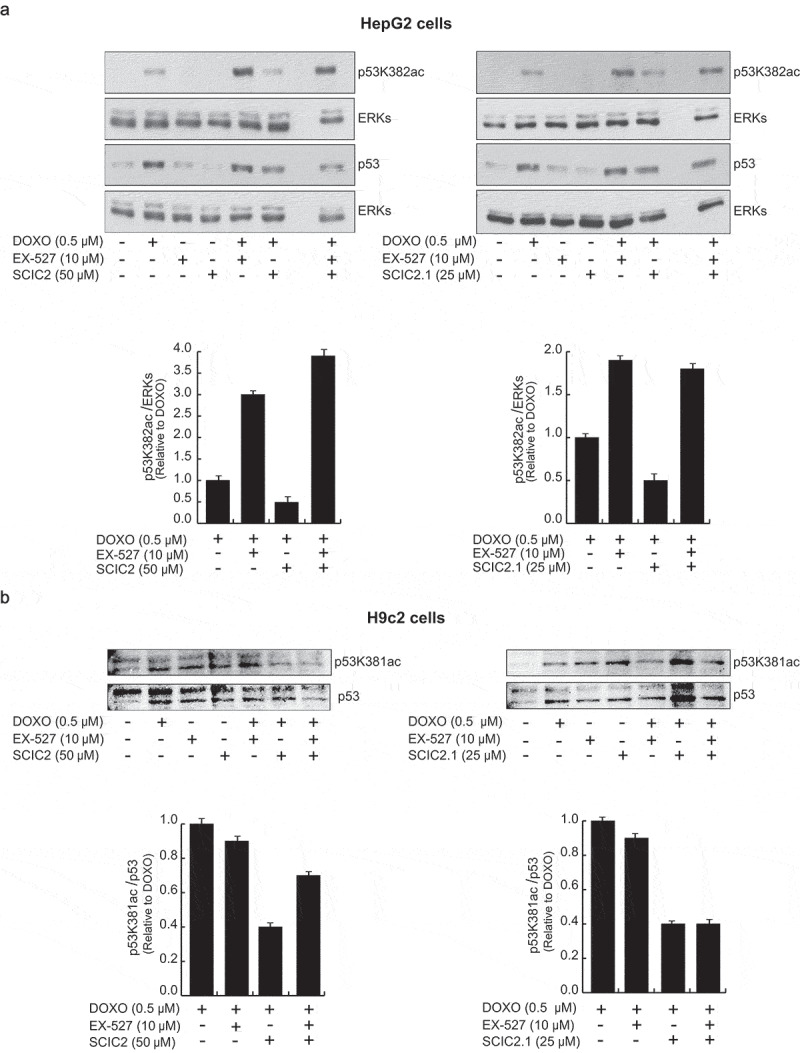
Effects of SCIC2 and SCIC2.1 on p53 acetylation. (a–b) Western blot analyses for p53 and p53K382/381ac from whole extracts of HepG2 and H9c2 cells treated with indicated compounds at indicated doses and times. Band quantification was performed using ImageJ software. Values are mean ± SD; experiments were performed in triplicate.

**Figure 8.**
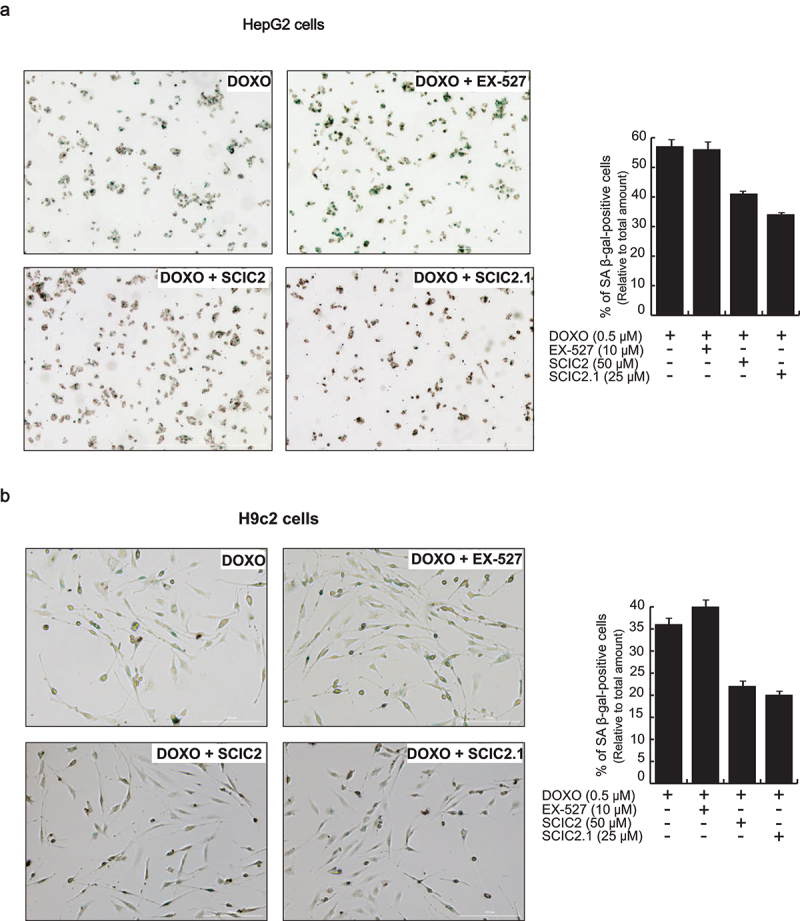
Effect of SCIC2 and SCIC2.1 on senescence in HepG2 and H9c2 cell systems. (a–b) Analysis of senescence-associated βgalactosidase (SA-β-gal) activity in HepG2 (a) and H9c2 (b) cells. Panels represent the ratio between positive (green) cells and total cells. Values are mean ± SD; experiments were performed in triplicate.

